# Application of Terahertz Radiation to Soil Measurements: Initial Results

**DOI:** 10.3390/s111009973

**Published:** 2011-10-21

**Authors:** Volker Dworak, Sven Augustin, Robin Gebbers

**Affiliations:** 1 Department Engineering for Crop Production, Leibniz-Institute for Agricultural Engineering, Max-Eyth-Allee 100, 14469 Potsdam, Germany; E-Mail: rgebbers@atb-potsdam.de; 2 German Aerospace Center (DLR), Institute for Planetary Research, Experimental Planetary Physics (XP), Rutherfordstr. 2, 12489 Berlin, Germany; E-Mail: sven.augustin@dlr.de

**Keywords:** THz, transmission measurement, soil sensor, soil absorption, imaging, buried objects, soil parameters

## Abstract

Developing soil sensors with the possibility of continuous online measurement is a major challenge in soil science. Terahertz (THz) electromagnetic radiation may provide the opportunity for the measurement of organic material density, water content and other soil parameters at different soil depths. Penetration depth and information content is important for a functional soil sensor. Therefore, we present initial research on the analysis of absorption coefficients of four different soil samples by means of THz transmission measurements. An optimized soil sample holder to determine absorption coefficients was used. This setup improves data acquisition because interface reflections can be neglected. Frequencies of 340 GHz to 360 GHz and 1.627 THz to 2.523 THz provided information about an existing frequency dependency. The results demonstrate the potential of this THz approach for both soil analysis and imaging of buried objects. Therefore, the THz approach allows different soil samples to be distinguished according to their different absorption properties so that relations among soil parameters may be established in future.

## 1. Introduction

Soil is a scarce resource which requires due attention. Most of our food and feed crops are grown on soils. Soils are important for recycling of nutrients and cleaning of groundwater by mineralization and they play a crucial role in climate change due to their capability to store and release greenhouse gases. Soils are formed by weathering of the upper layer of the Earth and consist of three phases (solid, liquid, gaseous). They are compositions of mineral, organic matter and soil organisms. Knowledge on soil parameters like carbon, water, and nitrogen content as well as particle size distribution is important for agriculture in particular and for environmental protection in general. However, conventional sample-based soil mapping is time-consuming and expensive while it requires large laboratory capacities. Facing the high heterogeneity of soil from the micro to landscape scale makes the situation even worse. Thus, many studies have been focused on mathematical methods to predict spatial soil variation by limited numbers of samples [1,2]. More recently, scientists have started to develop sensors for analyzing soil parameters *in situ*. When linked to global navigation satellite system these sensors should be able to map soil parameters quickly with a high spatial resolution at reasonable costs. Some sensors are already available for continuous mobile measurement, for example, galvanic coupled electrodes [3,4] or inductive coils [5,6] in a geo-electric regime, mechanical sensors for draft force measurements, spectrophotometers for optical properties [7,8], and probes for neutron scattering [9]. Other sensors are working in a stop-and-go mode, such as vertical penetrometers for soil compaction measurements [10–12]. The stop-and-go mode is also used for some dielectric measurements [13,14]. It has been demonstrated that the sensor-based approach for comprehensive measurements and micro variability analysis largely improved mapping results, even though each single sensor reading might be less accurate than a laboratory analysis and even when the sensors must be calibrated for each field [15]. Thus, this kind of proximal soil sensing seems to be most promising approach to improve land use and soil protection.

The use of terahertz (THz) electromagnetic waves is a completely new approach in the area of proximal soil sensing. Like radar, THz may serve as a nondestructive probe for soil analysis. The domain of THz technology is within 1 mm to 100 μm wavelength which separates it from ground-penetrating radar, working in the range of 300 m to about 15 cm. Whereas ground-penetrating radar is an established and still developing method to measure soil properties [16] the information derived from radar data is relatively limited. Radargrams of soils show contrasts in the dielectric properties. These are usually related to very coarse variations in the soil structure, like the occurrence of bedrocks, hardpans, and shallow water tables. To detect finer, more gradual variations of soils in a quantitative way the use of shorter wavelengths could be beneficial. This might be accomplished by THz technology. This technology is filling the gap between the traditional antenna applications and traditional optical applications with lenses. By applying both techniques for THz radiation, in particular the lens technique, we expect to obtain a higher spatial resolution than by radar. Additionally, THz technology may detect differences in soil composition based on soil depending attenuation, scattering and reflection. However, until now only very limited research was done on this kind of application. Thus, our study presents first attempts to make use of THz for soil analysis.

As a starting point, two THz sources with frequency range from 0.7 THz to 5.2 THz and 340 GHz to 360 GHz were available for the present study. These sources were used to discover if there are any frequency or soil sample-dependent signal variations. The establishment of a functional THz setup was complex and has been a major part in this study. High sensitive setups were needed, because the frequency dependence of water-absorption. Therefore, high and lower frequencies were tested to become less sensitive to water-absorption. There are water-absorption effects for lower frequencies, but the absorption is weak enough to allow high activities for measuring soil moisture with synthetic aperture radar [17–19]. In addition, frequencies ranging from 200 GHz to 400 GHz correspond to wavelengths ranging from 1.5 mm to 0.75 mm, which is comparable to the size of larger sand particles. This may cause scattering and absorption effects depending on the distribution of the soil particles. Frequencies from 340 GHz to 360 GHz were the focus of the present study.

The objectives of this study were to:

- establish an experimental setup and protocol for analyzing soil samples in the lab by THz radiation;
- determine attenuation coefficients for example soil samples;
- visualize attenuation contrasts by means of THz imaging.

If there are contrasts caused by different frequencies or soil samples, the THz approach could provide the opportunity to develop nondestructive soil sensors in the future.

## 2. Material and Methods

### 2.1. Soil Samples

Four different soil samples (Table 1) were used in order to estimate the absorption of THz radiation at different frequencies. They were selected with respect to variation in important soil parameters, namely organic matter (OM) and particle size distribution (soil texture) and with respect to the physical bulk density. Differences between the soil samples were important to induce contrasts for the THz approach. Three samples were natural soils collected in Potsdam, Germany (soils 1 to 3 in Table 1). One was an artificial soil, composed to obtain a sample with medium organic matter content (soil 4 in Table 1). The samples were ground, sieved to 2 mm and air dried. The air dried samples contained a certain amount of water as shown in Table 1, which may be relevant for THz attenuation. All samples were exposed to the same humidity conditions during the measurement campaign. They were analyzed for organic matter and water content (Table 1) according to VDLUFA [20–22].

**Figure 1.**
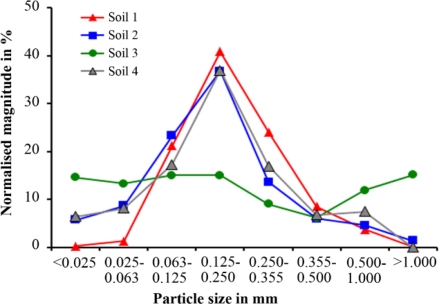
Particle size distribution of the four soil samples.

Particle size distribution was analyzed by sieving with different meshes. Figure 1 shows the distribution of particle sizes over eight classes.

Figure 1 shows a change in the tendency at particle sizes of 25 μm to 63 μm and 355 μm to 500 μm for all four soil samples. The smallest particle size was related to the amount of organic material in the soil samples (see OM in Table 1), except for Soil 4.

Bulk density of soils may be another influencing factor on THz attenuation. Therefore, bulk density was measured with a wedge sample holder and a measuring cup. Results of 10 repetitions are summarized in Table 2.

### 2.2. Transmission Measurements at 1.6 and 2.5 THz

For the evaluation of transmission greater than 1 THz, an optically pumped molecular gas laser was used. This laser operates at discrete frequencies between 0.7 THz and 5.2 THz, and it has an output power of up to 10 mW. The transmission measurements were done at frequencies of 1.627 THz and 2.523 THz. The measurements showed no sufficient transmission of THz radiation at this frequency range.

### 2.3. THz Setup for Determining the Absorption Coefficient

**Figure 2.**
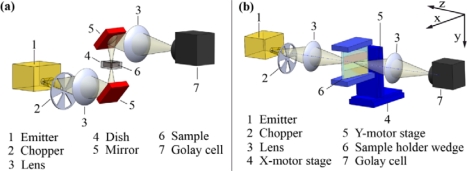
**(a)** Setup for the transmission measurement using the sample dish (approach 1). **(b)** Transmission measurement using the wedge sample holder (approach 2).

THz frequencies from 340 GHz to 360 GHz were generated with an yttrium iron garnet (YIG)-oscillator operating at 11–12 GHz as fundamental source. Multiplier diodes were used to upconvert the signal of the YIG oscillator (VDI-TX-S119, Virginia Diodes, Inc., Charlottesville, VA, USA). The power was emitted from a horn antenna with a Gaussian beam shape of 9° divergence at approximately 1 mW output power. The beam was optically modulated with a chopper wheel with frequencies ranging from 23 Hz to 27 Hz and then amplified by a lock-in amplifier. A lens made of TPX^®^ was used to focus the emitted power on the sample [Figure 2(a,b)]. A second lens was used to collect the power transmitted through the soil sample and to focus it onto a Golay cell detector (Tydex, St. Peterburg, Russia). The transmitted power was amplified with a lock-in amplifier (SR850, Stanford Research Systems, Inc., Sunnyvale, CA, USA), which in turn was referenced to the optical chopper. Data acquisition was done automatically with a computer.

**Figure 3.**
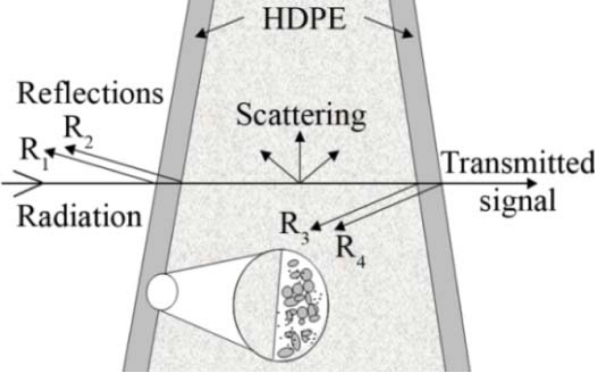
Wedge sample holder. The reflection coefficient of the surface to wall material interface is approximately constant by design.

Two setups were tested for exposing the samples to the THz radiation. In the first setup, the soil was placed in a Teflon sample dish. After putting the soil into the dish, the sample was carefully compacted, and its thickness was measured [Figure 2(a)]. The second setup employed a wedge sample holder of 6 cm height (y-direction), 6 cm length (x-direction) and of varying width from 0 cm to 2 cm (z-direction). The walls of the wedge sample holder were made of 2 mm high density polyethylene (HDPE) plates. This second setup was designed to reduce the complexity in determining the soil absorption coefficient. Based on the constant thickness of the walls and their plain surfaces absorption and refection coefficients of the wedge sample holder were independent from the position of the THz beam as shown in Figure 3. This was also true for the interface to the soil sample, because the diameter of the divergent beam was much larger than the soil particles.

The transmitted power received by the Golay cell detector, *Pt*, is given by:

(1) $$P_{t}(d)=P_{e}T_{\mathrm{atm}}T_{1}T_{2}e^{-\alpha d}$$

(2) $$\frac{P_{t}(d)}{P_{e}}=T_{\mathrm{atm}}T_{1}T_{2}e^{-\alpha d}$$

where *P_e_* is the power emitted from the source, *T_atm_* is the atmospheric transmission, *T*_1_, *T*_2_ are the transmission of the HDPE windows of the sample holder, *d* is the thickness of the soil at the measuring position and *α* is the absorption coefficient of the soil. The transmitted electromagnetic wave must pass all materials in series and therefore the transmission coefficients must be multiplied in Equation (1).

From this the absorption coefficient follows as:

(3) $$\text{ln}\left(\frac{P_{t}(d)}{P_{e}}\right)=\alpha d-\text{ln}(T_{\mathrm{atm}}T_{1}T_{2})$$

The atmospheric transmission and the transmission of the HDPE windows are constant and appear as an offset to the Equation (3). The Equation (3) explains why the linear regression can be used to determine the absorption coefficient and why the unknown reflection-coefficients do not affect the soil dependent absorption coefficient. The soil samples were compacted by knocking the wedge on a table. The soil density in the wedge varied from high to low from the bottom to the top of the wedge, caused by the soil’s own weight. The same protocol was applied to all soil samples. The wedge was scanned with a xy-positioning stage ([Figure 2(b)] (LIMES 90, OWIS GmbH, Staufen, Germany). The increment in x- and y-direction was set to 1 mm while the focus of the beam was adjusted to the wedge mid-position in the z-direction. Lock-in integration time at each grid point of the xy-array was 1 s. By this we obtained an image of 60 columns and 60 rows summing up to 360 pixels.

### 2.4. THz Setup for Visualization of Attenuation Contrasts (for Discrimination of Fresh Organic Matter and Mineral Soil Matrix)

**Figure 4.**
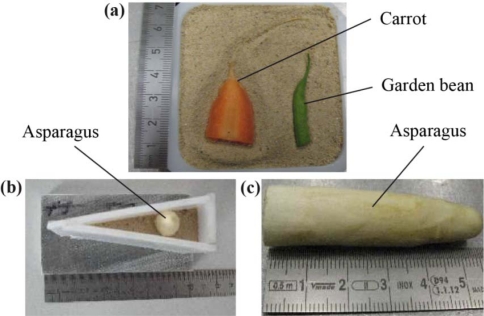
**(a)** Preparation of the carrot and garden bean; **(b** and **c)** Preparation of asparagus for the wedge sample holder.

**Figure 5.**
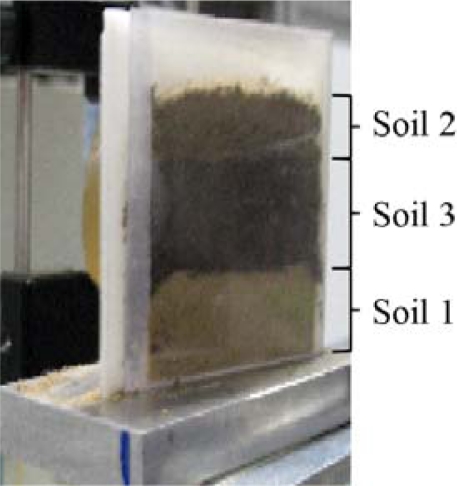
Wedge sample holder filled with three different soil samples. A transparent plastic wall material was used for the photo.

To investigate the ability of visualizing organic material with high water content embedded in air dried soils, a piece of a carrot as well as a garden bean were prepared in a dish (70 mm × 70 mm) [Figure 4(a)]. Possible signal variations caused by sample preparation, measurement day, and tilt of the wedge mount were minimized by filling the wedge with different soil samples at the same time. This layering of soils is shown in Figure 5. Additionally, a piece of asparagus was placed in the wedge sample holder as shown in [Figure 4(b,c)].

### 2.5. Determination of Absorption Coefficients

The absorption coefficients were derived from the wedge-scanning approach (second setup) by linear regression. Only data from the mid-region of the image were used. Data from the boarders were omitted due to distortion effects at the edges. Every column of data, collected along the y-direction, corresponded to a known, constant thickness of the sample. The sample thickness was 2 cm at the x-position (6 cm) for the wedge used, and so the absorption coefficient (in decibels per millimeter) needed to be multiplied by a factor of three. For regression analysis, data were averaged along the columns and then related to the respective sample thicknesses [Equation (4)]. Only those columns were used that have more than 20 values exceeding the least significant bit of the analog to digital converter. Linear regression models were fitted to the data by means of ordinary least squares. The slope of the regression line is a direct estimate of the absorption coefficient as expressed in decibels per millimeter. This can be explained as follows:

- No offset zeroing was needed for the absorption of the HDPE wedge wall material;
- No emitter amplitude adjustment was required (as long as the output power was stable during at least one scan);
- The absorption coefficient was given by the slope of the linear regression, thereby making it independent of the sample thickness. Therefore, the x-position did not need to be adjusted for the measurement series.

The following equations were used:

(4) $$\mathrm{mean}(x)=\frac{\sum_{i=1}^{n}I(x;y_{i})}{n}$$

(5) S(x)=10⋅log[mean(x)]

(6) S(x)=offset+αx/3

(7) d=x/3

where *I*(*x*;*y*) is the signal intensity at the xy-position in the image from the lock-in amplifier output, and *S*(*x*) are the mean values along each column (*y_i_*) on a decibel scale. At each day of measurement, temperature and humidity were recorded. All values, including the frequencies used, are listed in Table 3.

## 3. Results and Discussion

The analysis with frequencies of 1.627 THz and 2.523 THz show no transmission of detectable intensities. This setup had no options for separating scattering effects from damping effects, but both parameters could be high, because of the manifold composition of soil samples.

### 3.1. Evaluation of Different Experimental Setups

The small amount of soil sample area in the 25 mm × 25 mm dish caused poor repeatability among the reproductions of sample preparations (data not shown). The high fluctuations of the signal intensity are shown in Section 3.3. The main reasons for the poor repeatability included variations in the soil sample, an insufficient thickness preparation procedure with respect to the stamp and variations in the emitter power on different days. However, some differences for the transmitted signal intensity among the soil samples were detectable. The second approach that used the wedge sample holder was more efficient because all sample thicknesses were measured in one scan, and the influence of the soil density was averaged for the y-height. Therefore, the dish setup was only used with a 75 mm × 75 mm dish to image the buried test objects in the soil (Figure 4).

**Figure 6.**
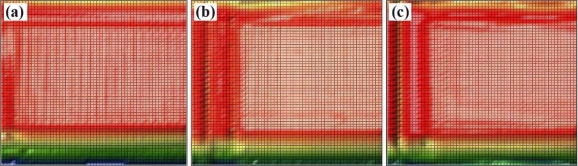
Reference measurement of the empty wedge sample holder with **(a)** 351 GHz, **(b)** 360 GHz and **(c)** 340 GHz in arbitrary units in rainbow colors from red, high intensity, to blue, low intensity. Each image size is 60 mm by 60 mm.

The wedge sample holder has the benefit that no position adjustments or calibrations are needed to determine the absorption coefficients. However, the usability of this setup must be evaluated. Therefore, the empty wedge sample holder was measured at three frequencies in order to estimate its influence on the transmitted signal intensity. For all three frequencies, an undistorted center region was visible (Figure 6).

The artifacts at the corners, as shown in Figure 6, are caused by the frame of the wedge holder, which shadowed parts of the divergent beam. The transmission at the three frequencies as measured in the central region of the holder is slightly different. However, this appears as an offset in the analysis and does not affect the attenuation coefficients of the soil samples.

### 3.2. Absorption Coefficients

**Figure 7.**
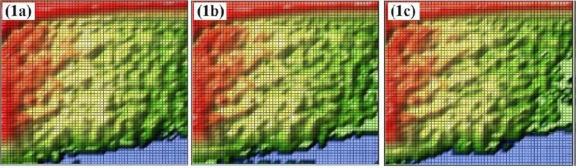

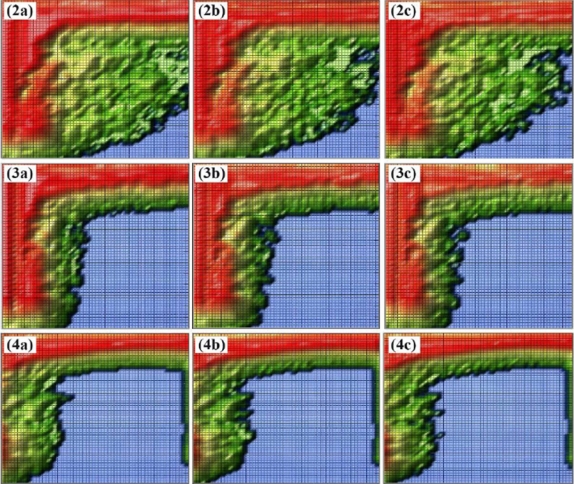
**(1a, 1b and 1c)** Transmission images of Soil 1 at 340 GHz, 351 GHz and 360 GHz. **(2a, 2b and 2c)** Transmission images of Soil 2 at 340 GHz, 351 GHz and 360 GHz. **(3a, 3b and 3c)** Transmission images of Soil 3 at 340 GHz, 351 GHz and 360 GHz. **(4a, 4b and 4c)** Transmission images of Soil 4 at 340 GHz, 351 GHz and 360 GHz. All images are in arbitrary units in rainbow colors from red, high intensity, to blue, low intensity. Each square in the images is equal to one square millimeter and the image size is 60 mm by 60 mm.

As shown in Table 3, nearly equal frequencies were used to measure the local transmitted signal intensity. The resulting images, shown in Figure 7, indicate why averaging along the y-direction was required. One can observe strong local signal variations. Thus, a single point measurement could be misleading and would not be adequate for characterizing these difficult soil samples.

**Figure 8.**
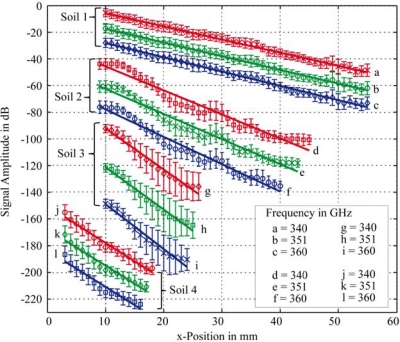
Plots of transmission signal amplitude versus x-position of the wedge sample holder for the four soil samples at three different frequencies (the x position is related to the soil thickness by d = x/3). All plots are adjusted with an additional offset for better visibility.

The blue regions in the acquired images (Figure 7) represent a measured intensity of zero, meaning that the signal intensity was below the detection limit of the setup in this configuration. Because zero cannot be a permissible value on the decibel scale, all zeros were rejected and replaced by missing values. The mean value and standard deviation for each column are plotted in Figure 8. Every plot was adjusted with an additional offset to separate the graphs and to increase visibility. Lengths of plots vary in Figure 8 for the different soil samples, because only those signal amplitudes were included that are based on more than 20 nonzero measurements in a column.

Figure 8 shows representative plots of the signal absorption for the four soil samples measured with three different frequencies. The results varied with the size and position of the used inner xy-array of the data points. Therefore, the estimated absorption coefficients are inaccurate as shown by the repeated measurements at 340 GHz in Table 4.

**Figure 9.**
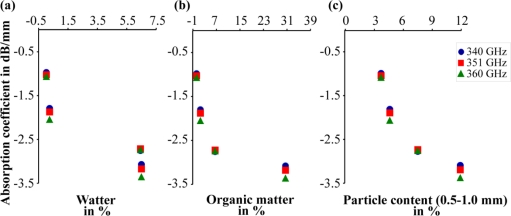
**(a)** Absorption coefficient versus the water content; **(b)** Absorption coefficient versus the organic matter; **(c)** Absorption coefficient versus the particle content of the 0.5–1.0 mm fraction.

Thus they should not be used for precise predictions. However, the differences between the absorption coefficients were obvious. These results demonstrated the feasibility of discriminating soils by THz radiation. With a more sensitive and stable setup as well as a mare reproducible sample preparation, quantitative measurements may be possible. A well defined calibration protocol is required. With respect to the error of estimate of the absorption coefficient (Table 4), higher absorption can be assumed with higher measuring frequencies for the first three soil samples (Table 4). This rule is not applicable to the THz analysis of the fourth soil sample. We may conclude that spectral information in the THz region contains additional information about different absorption mechanisms. To verify this in further experiments more stable measurements over a wider frequency range are required. The influence of water, carbon, nitrate and other soil ingredients needs to be analyzed in more detail. The amount of water and carbon increased in the same manner for the first three soil samples (Table 1). Therefore, the mechanism that dominates the absorption effect cannot be defined with this preparation [Figure 9(b,c)]. Moreover, the influence of the particle size distribution may be responsible for the different spectral behavior of the fourth soil sample. The first three soil samples had the same sequence for the value of the absorption coefficient and the amount of large and small particles, and the sequences were inversed for the middle particle size. Soil 4 had the same middle value as Soil 2, but they had a different distribution for small and large particle densities (Figure 1). The water content of Soil 3 and Soil 4 was nearly identical, and so water content was not the reason for the different spectral behavior [Figure 9(a)].

**Figure 10.**
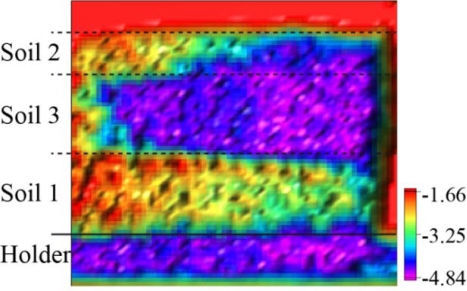
The transmission image of the first three soil samples with a logarithmic scale in arbitrary units. From top to bottom: Soil 2, Soil 3 and Soil 1. The image size is 60 mm by 60 mm.

With respect to the densities (Table 2) of the four soil samples, the signal absorption is not dominated by the density, because Soil 1 has the highest density but the lowest absorption. Therefore, the absorption is dominated by the conducting and dielectric properties of the soil ingredients and not by the density. As previously mentioned, one disadvantage of the setup in the present study is the poor reproducibility of the absorption coefficients. Table 4 shows the results of the first measurements and the three repeated measurements for 340 GHz. The difference between the measured results demonstrates the difficulties in reproducing the sample preparation and alignment. Humidity changes may have also caused the signal variations. However, humidity changes did not dominate the effect because humidity was higher for the second measurement of Soil 2 in which the determined absorption coefficient was lower. The difference for Soil 3 may have been caused or amplified by the restricted number of useful data points, resulting in unreliable quantitative results. The alignment difficulties may be overcome in the future with two modifications of the setup. First, implementing a snap-in adapter for the wedge sample holder may allow for higher reproducibility of the mechanical alignment, although the problem of refilling the sample holder in a reproducible manner would not be solved. However, this could be solved with a second modification by using a reference sample that has to be measured together with the soil sample in the wedge at the same time. These modifications should allow better quantitative measurements in future experiments. This new approach is demonstrated in Figure 10 with the first three soil samples. The beam divergence caused a triangle overlay in the signal between the soil samples. Therefore, a larger wedge sample holder or a smaller beam divergence is required.

### 3.3. Imaging

The ability to focus the THz beam has the advantage of a high spatial resolution of localized absorption causes, thus, enabling imaging. Visualizing with high spatial resolution in terms of images is an important application for a sensor setup. The potential of this visualization method is shown in Figure 10 with an image of three different soil samples in the wedge sample holder.

The different signal absorption of the three soil samples was visualized by the image colors and was highly detectable. The signal absorption in the bottom region was an edge effect from the overlay of the divergent beam and the aluminum part of the sample holder. Under this setup condition, the Lock in amplifier was overdriven for the non-soil region, allowing better signal resolution in the soil region. Thus, the signal amplitude was still detectable for the highest sample thickness.

The imaging capability of THz radiation was also demonstrated in the second approach. The ability to find and analyze buried vegetables is also an important instrumentation for horticulture, to find and analyze asparagus for example. The THz approach may also be a solution for the difficulties encountered in analyzing buried objects because of the high spatial resolution and soil penetration possibilities.

**Figure 11.**
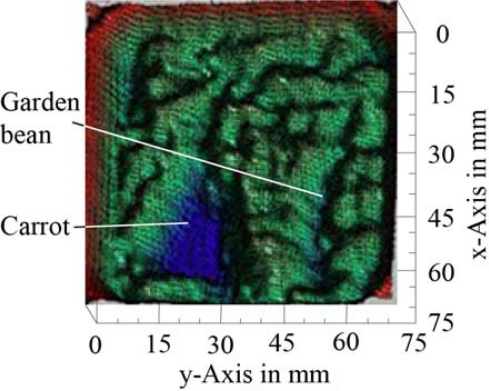
The transmission image of the dish sample holder filled with Soil 1, a piece of carrot and a garden bean [see Figure 4(a)]. The image is in arbitrary units in rainbow colors from red, high intensity, to blue, low intensity.

**Figure 12.**
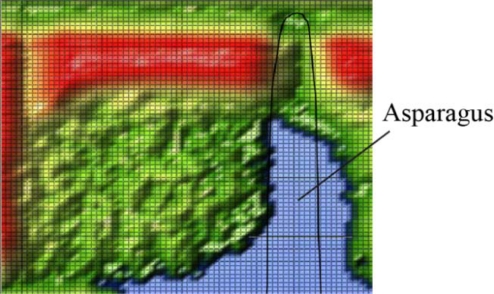
The transmission image of the wedge sample holder filled with Soil 1 and asparagus. The image is in arbitrary units in rainbow colors from red, high intensity, to blue, low intensity. The image size is 60 mm by 60 mm.

The high water content of vegetables caused signal absorption for the transmitted THz wave. The blue regions in Figure 11 indicate the positions of the carrot and garden bean. The garden bean had less signal absorption because of its smaller size and lower water content as compared to the carrot. The image in Figure 11 is overlaid with some type of interference, which demonstrates the poor absorption coefficient analysis under the dish approach. Figure 12 shows the absorption image of the asparagus prepared in the wedge sample holder [see also Figure 4(b,c)].

The position of the asparagus was localized using a higher signal absorption, and the hand-drawn dimension of the asparagus was manually added to Figure 12. The signal amplitude, dynamic range and beam divergence must be optimized for better spatial resolution and information content. However, the imaging capability of the THz approach was clearly demonstrated, thus suggesting advantages for future applications in horticulture experiments. High-oriented structures in the asparagus may also induce different signal absorption for different polarizations of the transmitting signal, which may be used to raise or insert a new contrast in the image in future applications.

## 4. Conclusions

Two experimental setups to analyze soils by THz electromagnetic waves were tested. One approach is based on a simple dish sample holder, the other is based on a wedge holder. Using the dish sample holder turned out to be difficult because of variations in filling, alignment and interference effects. These variations resulted in an inadequate degree of reproducibility, in the determination of the absorption parameters of soil samples. The frequency range from 340 GHz to 360 GHz had a penetration depth of more than 20 mm at a source power of 1 mW for soil samples with low water content or organic material. THz frequencies above 1.5 THz were not able to interfuse thin soil samples in a range of 1 mm with the setup used in this study. Therefore, a frequency dependency for the absorption coefficient over such a large frequency range is highly detectable. The available transmission of 350 GHz radiation allowed for the determination of soil-dependent absorption coefficients, but sample heterogeneity or preparation errors require adequate averaging. The wedge sample holder approach introduced here is an elegant way to solve this problem because the measured data can be averaged in the y-direction and additional the results in the x-direction, yielding direct throughput to absorption variations based on sample thickness. In this approach, complete measurement can be done without changing the setup. Moreover, it does not require any offset calibration of amplitudes or xy-positions, which is the main advantage of the wedge sample holder approach.

In this study, the feasibility of the THz imaging of soils was demonstrated. The local parameter variations of different soil samples were detectable. In addition, it was possible to differentiate different soil samples by their differing absorption coefficients (see Table 4 and Figure 9). Soils with higher organic matter have higher absorption coefficients. With respect to the corresponding wavelength of less than 1 mm particle size fraction of 0.5 mm to 1 mm shows higher absorption for higher content. However, the alignment of an optical THz system is difficult. For example, stationary waves and scattered emissions have to be avoided by careful design. The current setup can be further improved. Nevertheless, the practical outcome of this study is the establishment of an approach to perform soil analyses by THz radiation. With a more sensitive detector and better calibration, it may be possible to make a quantitative assessment of soil parameters. Furthermore, additional spectral information should help to separate the information overlay of different soil parameters. Even a third dimension can be added to the setup by varying the focus point mechanically or using a time-of-flight principle to detect distance information. It may be possible that a complete scan can be done electronically using a phase antenna array. This requires that the phase information is not obstructed by scattering and runtime effects in the soil. In this study, a soil layer of 2 cm was penetrated with a source power of just 1 mW. This suggests that soil layers of more than a few centimeters can be analyzed by a THz setup with more powerful signal sources.

Measurements need to be more accurate to establish the absorption coefficient in a quantitative manner. To solve accuracy issues, two options should be investigated in the future. First, a THz setup with more powerful signal sources, larger lenses and more sensitive detectors, such as a heterodyne amplifier should be employed. Second, measurements with a known and unknown soil samples should be carried out in tandem to improve prediction. This requires a larger wedge sample holder and smaller beam divergence.

## Figures and Tables

**Table 1.** Analysis of the air dried soil samples. The difference to 100% is the leftover which is so called “mineral ashes”.

**Sample name**	**DM105** %	**OM** %	**C** %	**N** %	**S** %
Soil 1	99.97	0.238	0.010	0.001	0.006
Soil 2	99.75	1.554	0.451	0.017	0.030
Soil 3	93.50	30.50	15.50	0.142	3.61
Soil 4	93.57	6.55	0.748	0.009	0.026

DM 105 is the dry matter of the sample after oven-drying for 24 h at 105 °C; OM is the amount of organic matter; C, N, and S are the concentrations of carbon, nitrogen, and sulfur, respectively.

**Table 2.** Bulk densities of the four soil samples.

**Container**	**Density in g/cm^3^**							
	**Soil 1**		**Soil 2**		**Soil 3**		**Soil 4**	
Wedge	1.7879	±0.0430	1.2860	±0.0315	1.0123	±0.0371	1.0511	±0.0241
Measuring cup	1.7937	±0.0274	1.3987	±0.0242	1.0031	±0.0151	1.0551	±0.0160

**Table 3.** Test procedure with the used measurement frequencies.

**Date**	**Sample**	**Frequency GHz**	**Room temperature °C**	**Humidity %**	**Plot in Section 3.2**
20.01.2010_1510	holder	351	20.5	21.8	
20.01.2010_1655	Soil 1	351	20.5	21.8	b
20.01.2010_1840	Soil 1	340	20.5	21.8	a
20.01.2010_2021	Soil 1	360	20.5	21.8	c

22.01.2010_1238	holder	340	20.8	17.1	
22.01.2010_1429	Soil 3	340	20.8	17.1	g
22.01.2010_1611	Soil 3	351	20.8	17.1	h
22.01.2010_1752	Soil 3	360	20.8	17.1	i

25.01.2010_1239	holder	360	20.1	13.2	
25.01.2010_1441	Soil 2	360	20.1	13.2	f
25.01.2010_1622	Soil 2	351	20.1	13.2	e
25.01.2010_1802	Soil 2	340	20.1	13.2	d

26.01.2010_1335	Soil 4	340	20.0	11.7	j
26.01.2010_1515	Soil 4	351	20.0	11.7	k
26.01.2010_1657	Soil 4	360	20.0	11.7	l

02.02.2010_1213	holder	340	21.4	22.5	
02.02.2010_1400	Soil 1	340	21.4	22.5	
02.02.2010_1552	Soil 2	340	21.4	22.5	
02.02.2010_1810	Soil 3	340	21.4	22.5	

**Table 4.** Absorption coefficient estimation with linear regression.

**Freq GHz**	**Soil 1**		**Soil 2**		**Soil 3**		**Soil 4**	

	**D** **dB mm^−1^**	**R^2^**	**D** **dB mm^−1^**	**R^2^**	**D** **dB mm^−1^**	**R^2^**	**D** **dB mm^−1^**	**R^2^**
340	−0.98 ± 0.02	0.996	−1.80 ± 0.124	0.961	−3.08 ± 0.271	0.975	−2.76 ± 0.21	0.983
351	−1.03 ± 0.022	0.995	−1.88 ± 0.115	0.971	−3.18 ± 0.278	0.977	−2.72 ± 0.291	0.969
360	−1.07 ± 0.023	0.995	−2.05 ± 0.132	0.971	−3.36 ± 0.377	0.966	−2.74 ± 0.4	0.949
340	−1.02 ± 0.031	0.991	−1.42 ± 0.088	0.981	−3.82 ± 0.284	0.996		
